# Editorial: Children and adolescent health-related behaviors

**DOI:** 10.3389/fpubh.2023.1263452

**Published:** 2023-07-31

**Authors:** Maha El Tantawi

**Affiliations:** Faculty of Dentistry, Alexandria University, Alexandria, Egypt

**Keywords:** adolescents, health behavior, physical activity, tobacco use, behavior modification

The United Nations Convention on the Rights of the Child (1) recognizes the right of the child to the highest attainable standard of health. A life course approach to health acknowledges the importance of exposures and experiences in early life to the health and wellbeing of individuals in later stages of their life. The health of adolescents and children impacts their health in later life and the health habits they develop in early life affect their health in the long run (2). The Sustainable Development Goals (SDGs) also aim to ensure that every child survives and thrives (3) and acknowledges that children wellbeing is critical to the achievement of sustainable development. Greater focus is needed on the health of children and adolescents and associated determinants to establish healthcare programs tailored to their needs and characteristics and to allocate more resources to support them. This Research Topic provides this focus.

The Research Topic sheds light on risk factors that may impact young people's health like they impact the health of adults such as use of tobacco, obesity, inadequate sleep and limited physical activity in addition to risk factors that are more relevant to younger age groups such as longer screen use time and use of e-cigarettes. The quality of sleep and physical activity affect perceived and actual health. In this Research Topic, Ding et al. showed that short sleep periods and low sleep quality were associated with suboptimal self-reported health among medical students in China. Qin et al. reported an association between weight and physical fitness in Chinese high school students thus showing an association between physical activity and obesity in younger age groups. Physical activity has direct and indirect benefits for health. In this Research Topic, Zeng et al. showed that physical activity mitigated the negative impact of prolonged use of screens on visual acuity among Chinese children during the COVID-19 pandemic, thus, providing evidence to guide parenting practices. Despite this health promoting effect of physical activity, Deng and Fan presented data from a national US survey of adolescents showing that sports participation has declined in the last decade with differences among ethnic subgroups and subsequent negative health effects to be expected among this young population. Obesity (4) and sedentary lifestyles (5) are modern times pandemics in various age groups and if these problems currently observed in children and adolescents are not addressed, the burden of non-communicable diseases associated with this lifestyle can only be expected to increase.

Tobacco use is another global health problem with statistics showing that although cigarette smoking has decreased among adolescents, the use of other tobacco products has increased or remained the same over the last two decades (6). This Research Topic confirms the important effect of the social environment on young people's intended or actual tobacco use (7). Parents, siblings and friends who smoke facilitate smoking for youngsters. The Theory of Planned Behavior (8) posits that perceived norms are among the factors affecting intention to engage in a health behavior. How people surrounding an individual behave sets the stage for expectations and accepted standards. In this Research Topic, Dai et al., showed that exposure to second-hand smoking and multiple sales sources, as well as having parents and friends who use e-cigarettes were all associated with greater likelihood of Chinese adolescents' expressing an intention to use e-cigarettes. Mai et al. also confirmed the association between friends' using e-cigarettes and Chinese adolescents' use of e-cigarettes although they showed that some personality traits, like agreeableness, may be associated with lower odds of e-cigarettes' use. Ribera-Osca et al. also confirmed the impact of having parents who are smokers on secondary school students' tobacco use in Spain.

The Research Topic offers an insight into health intervention opportunities that are of special importance to children and adolescents by describing several school-based health interventions. School health programs offer a good chance to integrate health promotion activities into school activities and to reduce workforce needs by utilizing schoolteachers to supervise program activities. Gasoyan et al. described a program to improve oral health and reduce caries among Armenian schoolchildren in rural areas where fluoridation is not possible. Nagy-Pénzes et al. reported on a school-based program that improved health-related knowledge, reduced unhealthy eating and alcohol consumption and improved physical activity among Hungarian secondary school students. Gross et al. used quality improvement and participatory approach to design a health education curriculum to improve American adolescents' health behaviors. These various interventions demonstrate the usefulness of school-based interventions to modify and instill positive health behaviors thus benefiting from the social environment in which children and adolescents live and turning perceived norms and role modeling into an opportunity rather than a threat.

The health of children and adolescents is at a critical stage. New challenges specific to this age group and risks that are characteristic of older age groups but apply to younger populations require innovative solutions that build on the specific attributes of young people and their vulnerability to the impact of peers and role models. Involving members of the target group in designing health behavior modification interventions is key to promoting the health of children and adolescents.

## Author contributions

ME: Writing—original draft, Writing—review and editing.

## References

[1] United Nations. Convention on the Rights of the Child | OHCHR. Available online at: https://www.ohchr.org/en/instruments-mechanisms/instruments/convention-rights-child (accessed July 19, 2023).

[2] World Health Organization. Child and Adolescent Health Policy. Available online at: https://www.who.int/europe/teams/policy-and-governance-for-health-through-the-life-course/child-and-adolescent-health-policy (accessed July 19, 2023).

[3] UNICEF. SDGs for Children. Available online at: https://data.unicef.org/sdgs/ (accessed July 19, 2023).

[4] World Obesity Federation. World Obesity Atlas 2023. Available online at: https://www.worldobesity.org/resources/resource-library/world-obesity-atlas-2023 (accessed July 19, 2023).

[5] World Health Organization. Global Status Report on Physical Activity 2022. (2022) 1–112. Available online at: https://www.who.int/teams/health-promotion/physical-activity/global-status-report-on-physical-activity-2022 (accessed July 19, 2023).

[6] Ma C Xi B Li Z Wu H Zhao M Liang Y. Prevalence and trends in tobacco use among adolescents aged 13-15 years in 143 countries, 1999-2018: findings from the Global Youth Tobacco Surveys. Lancet Child Adolesc Health. (2021) 5:245–55. 10.1016/S2352-4642(20)30390-4 33545071

[7] Villanti A Boulay M Juon HS. Peer, parent and media influences on adolescent smoking by developmental stage. Addict Behav. (2011) 36:133–6. 10.1016/j.addbeh.2010.08.018 20855170

[8] Ajzen I. The theory of planned behaviour: reactions and reflections. Psychol Health. (2011) 26:1113–27. 10.1080/08870446.2011.613995 21929476

